# The imbalance between Bregs, Tfh, and Tregs in patients with anti-N-methyl-D-aspartate receptor encephalitis

**DOI:** 10.1007/s10072-023-06624-z

**Published:** 2023-02-13

**Authors:** Yatong Li, Jing Zhang, Lei Liu, Shilei Cui, Houliang Sun, Hanqiu Jiang, Yanjun Guo, Jingxiao Zhang, Zhuxiao Xie, Jiawei Wang

**Affiliations:** 1 Department of Neurology, Beijing Tongren Hospital, Capital Medical University, Beijing, China; 2 Clinical Research Center, Beijing Tongren Hospital, Capital Medical University, Beijing, China

**Keywords:** Anti-N-methyl-D-aspartate receptor encephalitis, Bregs, Tregs, Tfh

## Abstract

**Objective:**

To detect the alteration of regulatory B cells (Bregs), follicular helper T cells (Tfh), and regulatory T cells (Tregs) frequencies in patients with anti-N-methyl-D-aspartate receptor (anti-NMDAR) encephalitis. Analyze their association with clinical severity and activity, and explore the effects of different immunotherapies on those immune cell subsets.

**Methods:**

We enrolled 21 patients with anti-NMDAR encephalitis, 22 patients with neuromyelitis optica spectrum disorder (NMOSD), 14 patients with idiopathic intracranial hypertension (IIH), and 20 healthy controls (HC) in our study. The frequencies of various immune cell subsets were determined using flow cytometry.

**Results:**

Compared to patients with IIH and HC, the frequencies of CD24^hi^CD38^hi^ transitional B cells as well as Tregs were significantly lower while the frequency of Tfh was significantly higher in patients with anti-NMDAR encephalitis. The frequency of CD24^hi^CD38^hi^ transitional B cells was significantly lower in the acute stage than in the recovery stage, and was negatively correlated with the modified Rankin scale (mRS) and the clinical assessment scale for autoimmune encephalitis (CASE). The frequency of CD24^hi^CD38^hi^ transitional B cells at the last follow-up after rituximab (RTX) treatment was significantly higher than those treated with oral immunosuppressants or untreated. There was no clear difference between anti-NMDAR encephalitis and NMOSD in the above immune cell subsets.

**Conclusion:**

We suggested that the frequencies of CD24^hi^CD38^hi^ transitional B cells and Tregs were decreased while the frequency of Tfh was increased in patients with anti-NMDAR encephalitis. CD24^hi^CD38^hi^ transitional B cells frequency may be a potential indicator to estimate the disease activity and severity.

## Introduction

Anti-N-methyl-D-aspartate receptor (NMDAR) encephalitis is the most common type of autoimmune encephalitis (AE), accounting for about 80% of AE [1]. It is an autoimmune neuropsychiatric disorder that can cause a variety of clinical manifestations, including psychiatric disturbances, cognitive impairments, seizures, movement disorders, autonomic dysfunction, hypoventilation, and even coma [2]. There are two main triggers of anti-NMDAR encephalitis: tumors, usually teratomas, and central nervous system infections, such as herpes simplex encephalitis. B and T cells have been proposed to be involved in anti-NMDAR encephalitis. The potential pathogenesis is that the NMDAR, which is expressed in tumor nervous tissue or on the surface of neurons, may be released and handed over to the immune system. Memory B cells and plasma cells are produced in local lymph nodes and then pass through the blood–brain barrier (BBB). After undergoing a series of antigen-driven changes, memory B cells differentiate into mature plasma cells and secrete antibodies [3].

T cells and B cells homeostasis is disturbed in anti-NMDAR encephalitis. B cells play an important role in immunoregulation, both pro- and anti-inflammatory. Numerous studies have demonstrated that pro-inflammatory B cell phenotypes and cytokines were involved in pathogenesis of anti-NMDAR encephalitis [4–8]. However, it is still unknown whether the anti-inflammatory B cell phenotypes is also involved. Regulatory B cells (Bregs) are immunosuppressive cells that support immunological tolerance via the release of interleukin-10 (IL-10), IL-35, and transforming growth factor β (TGF-β) [9]. Bregs can suppress the proliferation and differentiation of effector T cells including T follicular helper cells (Tfh) and increase the number of regulatory T cells (Tregs) [10, 11]. In human, the two main Bregs subsets have been identified as CD24^hi^CD38^hi^ transitional B cells and CD24^hi^CD27^+^ B10 B cells [12]. Tfh is a newly discovered CD4^+^ effector T cell subset, characterized by surface marker expression of the chemokine receptor CXCR5, costimulatory molecules ICOS and PD-1. Tfh are mainly located in the germinal center (GC) of secondary lymphoid tissue and play an essential role in providing help for GC formation, B cell differentiation into plasma cells and memory cells, and antibody production. Dysfunction of Tfh may result in autoimmune diseases [13]. Regulatory T cells (Tregs), characterized as CD4^+^CD25^+^FoxP3^+^, is a specific suppressor subtype of CD4^+^ T cells and play a critical role in the maintenance of peripheral immune tolerance. Numerical, functional, or migratory deficits in Tregs may break self-tolerance and lead to autoimmune diseases [14].

Recently, an increasing number of studies have confirmed that quantitative or functional impairment of Bregs and Tregs, or deregulation of Tfh, is related to many neurological autoimmune diseases, such as multiple sclerosis (MS), neuromyelitis optica spectrum disorders (NMOSD), myelin oligodendrocyte glycoprotein antibody disease (MOG-AD), and myasthenia gravis (MG) [14–20]. However, the changes in Bregs, Tfh, and Tregs have not been investigated in anti-NMDAR encephalitis. Therefore, we detected the frequencies of Bregs, Tfh, and Tregs in peripheral blood in patients with anti-NMDAR encephalitis, and analyzed the frequencies of Bregs, Tfh, and Tregs in patients with different clinical stages and their correlation with the modified Rankin scale (mRS) and the clinical assessment scale for autoimmune encephalitis (CASE). Besides, we explored the effects of different therapies on Bregs, Tfh, and Tregs.

## Materials and methods

### Patients and controls

A total of 21 patients with anti-NMDAR encephalitis fulfilling the diagnostic criteria of Graus et al. published in 2016 [21] were recruited in the Department of Neurology, Beijing Tongren Hospital, Capital Medical University from September 2021 to October 2022. The patients’ diagnoses were all confirmed based on clinical manifestations and detection of anti-NMDAR antibodies in cerebrospinal fluid (CSF) samples via cell-based assays (CBA). The exclusion criteria were previous intravenous methylprednisolone pulse (IVMP) therapy within 1 month before sampling, any infection within 1 month before sampling, or coexisting cancer. Patients were permitted to receive oral immunosuppressants including corticosteroids and/or mycophenolate mofetil (MMF), intravenous immunoglobulin (IVIG), or rituximab (RTX) considering their condition.

For comparison, 22 patients with neuromyelitis optica spectrum disorder (NMOSD) and 14 patients with idiopathic intracranial hypertension (IIH) were recruited as other inflammatory neurologic disease (OIND) group and other noninflammatory neurological disease group (ONIND) respectively. Twenty age- and sex-matched healthy volunteers without organic diseases were recruited as the healthy controls (HC) group.

The study was approved by the local institutional review boards, and all participants (or their legal guardians) provided informed consent.

### Clinical assessment

It has been proposed that anti-NMDAR encephalitis could be divided into 4 stages [22]: prodrome and initial psychiatric symptoms stage, neurologic complications stage, recovery stage, cognitive, and behavioral sequelae stage. We defined the first two stages as the acute stage, while the other two stages as the recovery stage.

Neurological status was assessed with the mRS and the CASE. The mRS consists of six grades (0–5 points), and predominantly assess the impact of motor deficits on functional independence. While, the CASE consists of nine items, including seizure (current time), memory dysfunction, psychiatric symptoms (delusion, hallucination, disinhibition, aggression), consciousness, language problem, dyskinesia/dystonia, gait instability and ataxia, brainstem dysfunction, and weakness. Each item was assigned a point of 0–3, with exception of the item “brainstem dysfunction,” which consisted of gaze paresis, tube feeding, and ventilator care due to hypoventilation. The total scale ranged from 0 to 27.

### Sample collection and flow cytometry analysis

**Fig. 1:**
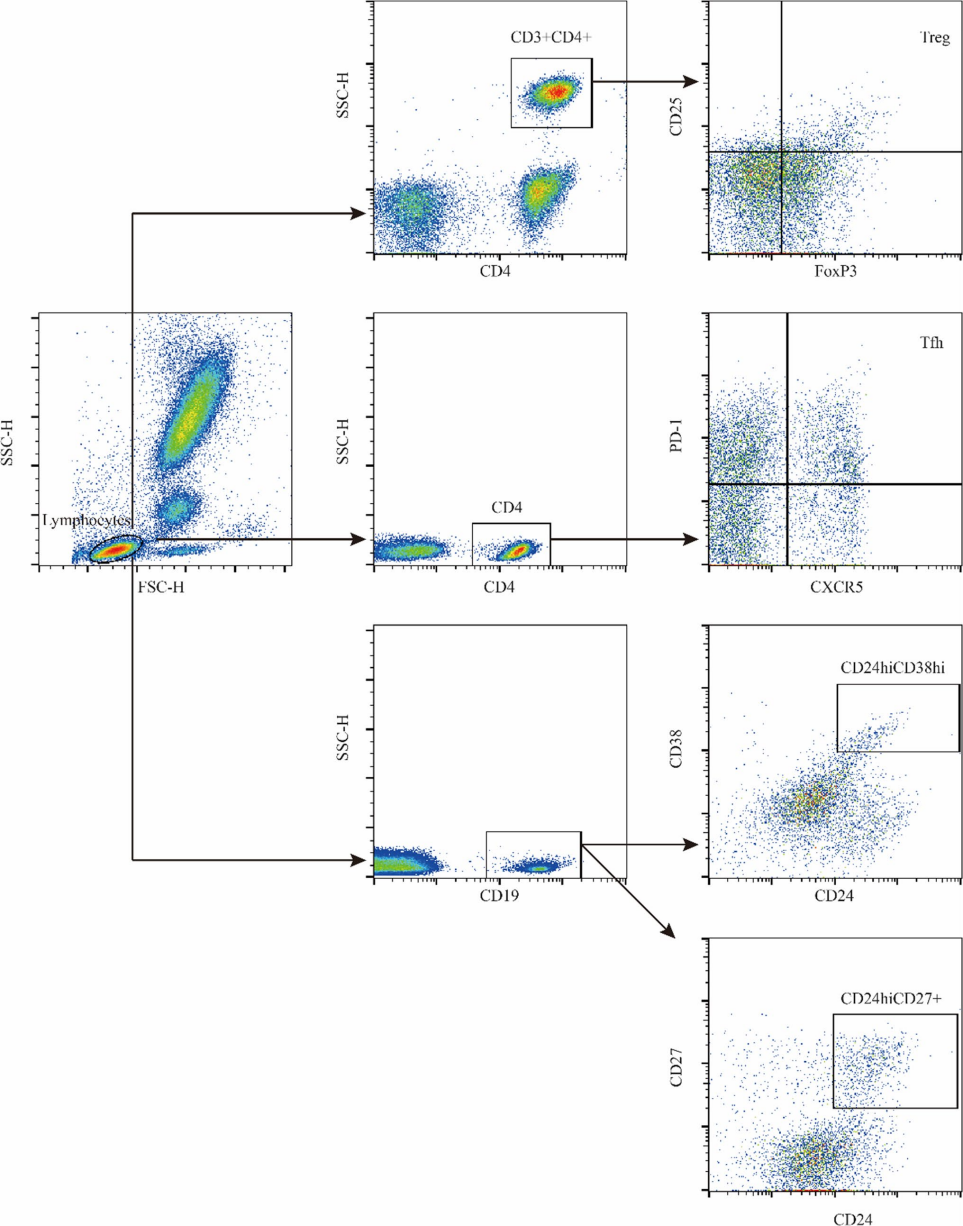
Gating strategies for Bregs, Tfh and Tregs. Lymphocytes were gated using forward scatter and side scatter, then, different gate settings were used to distinguish different immune cell subset. Tregs: CD3^+^CD4^+^ T cells were selected in lymphocytes, Tregs were identified as CD25 and FoxP3 double-positive cells. Tfh: CD4^+^ T cells were selected in the lymphocytes, Tfh were identified as PD-1 and CXCR5 double-positive cells. Bregs: CD19^+^ B cells were selected in the lymphocytes, Bregs were identified as CD24^hi^CD38^hi^ transitional B-cell and CD24^hi^CD27^+^ B10 B cell

Up to 2ml of peripheral blood samples were collected in EDTA anticoagulant tubes, stored at 4°C and processed for flow cytometry analysis within 24 h. The first tube was used for Bregs analysis. We analyzed two classical Bregs subsets: CD24^hi^CD38^hi^ transitional B cells and CD24^hi^CD27^+^ B10 B cells. One hundred microliters of EDTA anticoagulant blood were incubated with anti-human CD19-APC (clone HIB19), CD24-PE (clone ML5), CD27-PerCP-Cy5.5 (clone M-T271), CD38-FITC (clone HIT2), or corresponding isotype controls for 15min at room temperature and was protected from light. Then, treated with 10×FACS Lysing solution for 10 min. After washing twice in phosphate-buffered saline (PBS), samples were acquired. Lymphocytes were gated using forward scatter and side scatter, B cells were identified as CD19^+^ and then Bregs were identified as CD24^hi^CD38^hi^ transitional B cell and CD24^hi^CD27^+^ B10 B cells. The second tube was used for Tfh analysis. One hundred microliters of EDTA anticoagulant blood were incubated with anti-human CD4-FITC (clone RPA-T4), PD-1-PE (clone EH12.1), CXCR5-PerCP-Cy5.5 (clone RF8B2), or corresponding isotype controls. After lysing erythrocytes and washing, samples were acquired. CD4^+^ T cells were selected in the lymphocytes, and Tfh were identified as PD-1 and CXCR5 double-positive cells. The third tube was used for Tregs analysis. One hundred microliters of EDTA anticoagulant blood were incubated with anti-human CD3-PE-cyanine7 (clone HIT3a, BioLegend), CD4-FITC (clone RPA-T4), CD25-APC (clone M-A251), or corresponding isotype controls. After lysing and washing, samples were incubated with Fix/Perm buffer for 45min at 4°C, and then washed in perm/wash buffer. Samples were incubated with anti-human FoxP3-PE (clone 259D/C7) or corresponding isotype controls for 1 hour at 4°C. After washing, samples were acquired. CD3^+^CD4^+^ T cells were selected in lymphocytes, and Tregs were identified as CD25 and FoxP3 double-positive cells. All reagents were from BD Bioscience except where noted otherwise. Gating strategies were shown in Fig. 1.

Flow cytometry was performed on FACS Calibur (BD Biosciences). Single-stained cells were used to calculate compensation, and fluorescence minus one (FMO) controls were used to determine gating placement. Data were analyzed using FlowJo 10.8.1.

### Statistical analysis

All results were statistically analyzed by SPSS 26.0 and Graphpad Prism 8.0.2. Categorical variables were described by counts and percentages, while non-normal distributed continuous were described by medians and ranges. Fisher’s exact test was used for comparisons between frequency of categorical variables. Mann–Whitney *U* test was used for pairwise comparisons. Kruskal-Wallis test with Bonferroni post hoc analyses was used to compare multiple groups of samples. The Spearman rank correlation test was used to analyze correlations between parameters. A *P*-value <0.05 was considered statistically significant.

## Result

### Demographic and clinical features of anti-NMDAR encephalitis patients

The demographic data derived from patients and healthy controls was shown in Table 1. A total of 21 patients with anti-NMDAR encephalitis were included in our study. Five of whom were sampled after receiving RTX and formed the RTX group. The median interval between the last RTX infusion and sampling was 11 months (range 3–14). The remaining 16 patients who did not receive RTX formed the non-RTX group, 11 of whom were sampled while taking oral immunosuppressants (4 patients taking MMF, 7 patients taking corticosteroids plus MMF), and the others were untreated. Seven of 22 patients with NMOSD were sampled while taking oral immunosuppressants (4 patients taking MMF, 3 patients taking corticosteroids plus MMF), and the others were untreated. The proportion of patients taking oral MMF or corticosteroids plus MMF between no-RTX group and NMOSD group were comparable (*p*=0.429, *p*=0.176). None of the patients with IIH received immunosuppressants. There were no statistically significant differences among groups regarding age, gender, and disease duration (*p*=0.451, *p*=0.470, *p*=0.371)

**Table 1:** Demographic data of patients in the anti-NMDAR encephalitis, NMOSD, IIH, and HC groups

	Anti-NMDAR encephalitis		NMOSD	IIH	HC	*p*
	Non-RTX group	RTX group				
Total number of subjects	16	5	22	14	20	/
Demographic data						
Age (years)	32 (15–61)	28 (16–30)	34 (18–63)	30 (19–56)	29 (22–50)	0.451
Gender (male/female)	8/8	3/2	6/16	4/10	7/13	0.470
Disease duration (months)	14 (0.5, 40)	13 (4, 32)	13 (0.5, 42)	3 (0.5, 30)	/	0.371
Clinical data						
mRS	1.5 (0–4)	1 (1–2)	/	/	/	
CASE	3 (0–8)	2 (1–3)				
Abnormal behavior	13 (81.25%)	5 (100.00%)	/	/	/	
Memory deficit	6 (37.50%)	2 (40.00%)	/	/	/	
Seizure	11 (68.75%)	2 (40.44%)	/	/	/	
Movement disorder	1 (6.25%)	0	/	/	/	
Consciousness impairment	0	0	/	/	/	
Autonomic dysfunction	0	0	/	/	/	
Hypoventilation	0	0	/	/	/	
Treatment within 1 month before sampling						
Oral MMF	7 (43.75%)	/	4 (18.18%)	/	/	0.429
Oral corticosteroids plus MMF	4 (25.00%)	/	3 (13.64%)	/	/	0.176
Interval between last RTX infusion and sampling	/	11 (3–18)	/	/	/	

*mRS*, modified Rankin scale, *CASE*, the clinical assessment scale for autoimmune encephalitis, *MMF*, mycophenolate mofetil

Data are presented as counts (percentages) or medians (ranges)

### CD24^hi^CD38^hi^ transitional B cells, Tfh, and Tregs were imbalanced in patients with anti-NMDAR encephalitis

**Fig. 2:**
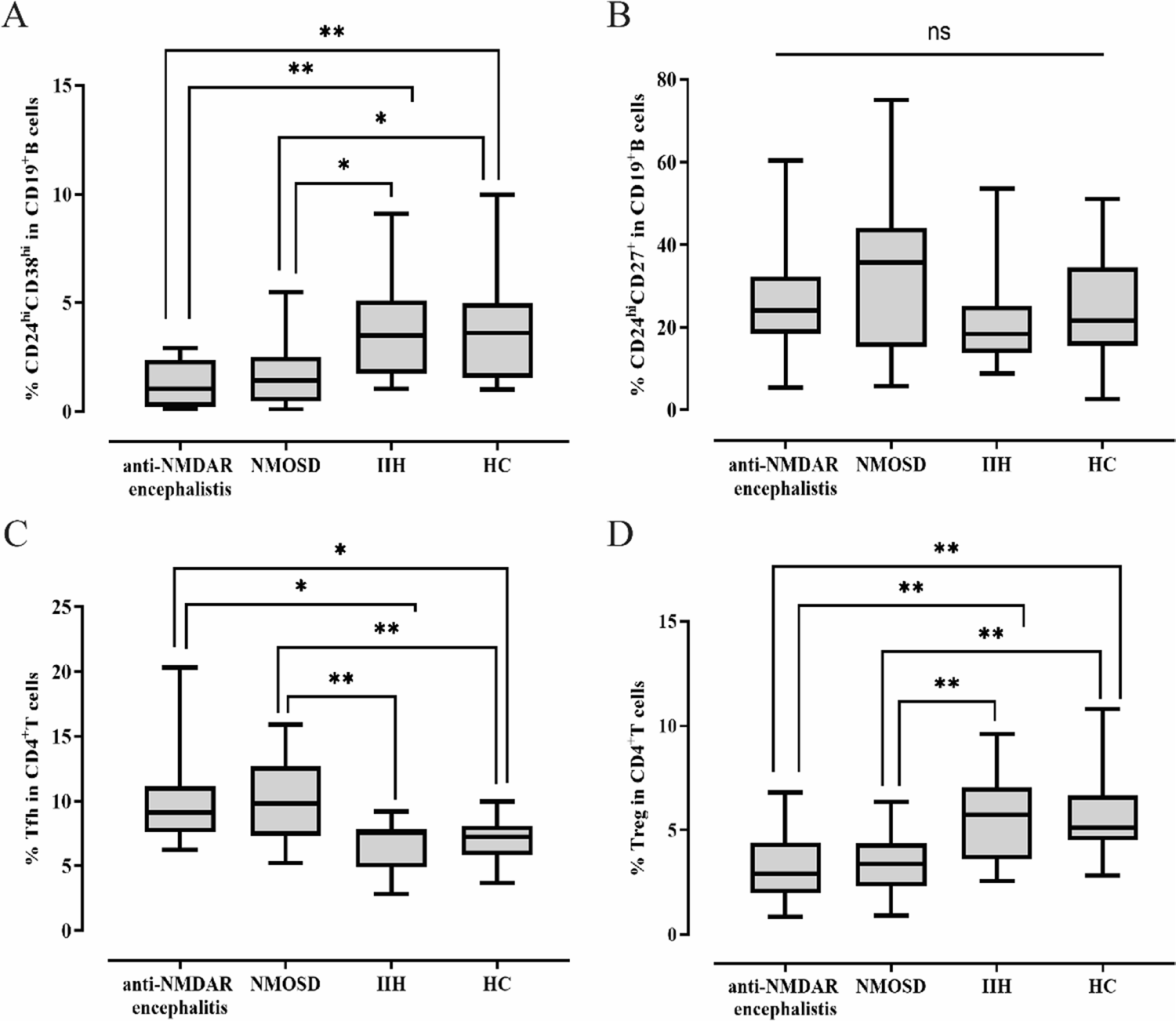
The comparisons of Bregs, Tregs, and Tfh in patients with anti-NMDAR encephalitis and controls. **A** Comparison of the frequency (as the percentage of CD19^+^B cells) of CD24^hi^CD38^hi^transtional B cells. **B** Comparison of the frequency (as the percentage of CD19^+^B cells) of CD24^hi^CD27^+^ B10 B cells. **C** Comparison of the frequency (as the percentage of CD4^+^T cells) of Tfh. **D** Comparison of the frequency (as the percentage of CD4^+^T cells) of Tregs. Kruskal-Wallis test with Bonferroni post hoc analyses was used. ^*^p<0.05, ^**^p<0.01

First, we compared the frequencies of peripheral CD24^hi^CD38^hi^ transitional B cells in CD19^+^B cells, CD24^hi^CD27^+^ B10 B cells in CD19^+^B cells, Tfh in CD4^+^T cells, and Tregs in CD4^+^T cells between anti-NMDAR encephalitis, NMOSD, IIH, and HC. As shown in Fig. 2, the frequencies of CD24^hi^CD38^hi^ transitional B cells and Tregs were significantly decreased and the frequency of Tfh was significantly increased in anti-NMDAR encephalitis when compared with IIH (*p*=0.007, *p*=0.003, *p*=0.025, respectively) and HC (*p*=0.004, *p*=0.001, *p*=0.033, respectively). However, the difference was not statistically significant when compared with NMOSD (*p*=1.000, *p*=1.000, *p*=1.000, respectively). There was no statistically significant difference in the frequency of CD24^hi^CD27^+^ B10 B cells in CD19^+^B cells between anti-NMDAR encephalitis, NMOSD, IIH, and HC (*p*=0.165).

### Change in CD24^hi^CD38^hi^ transitional B cells at different stages

**Fig. 3:**
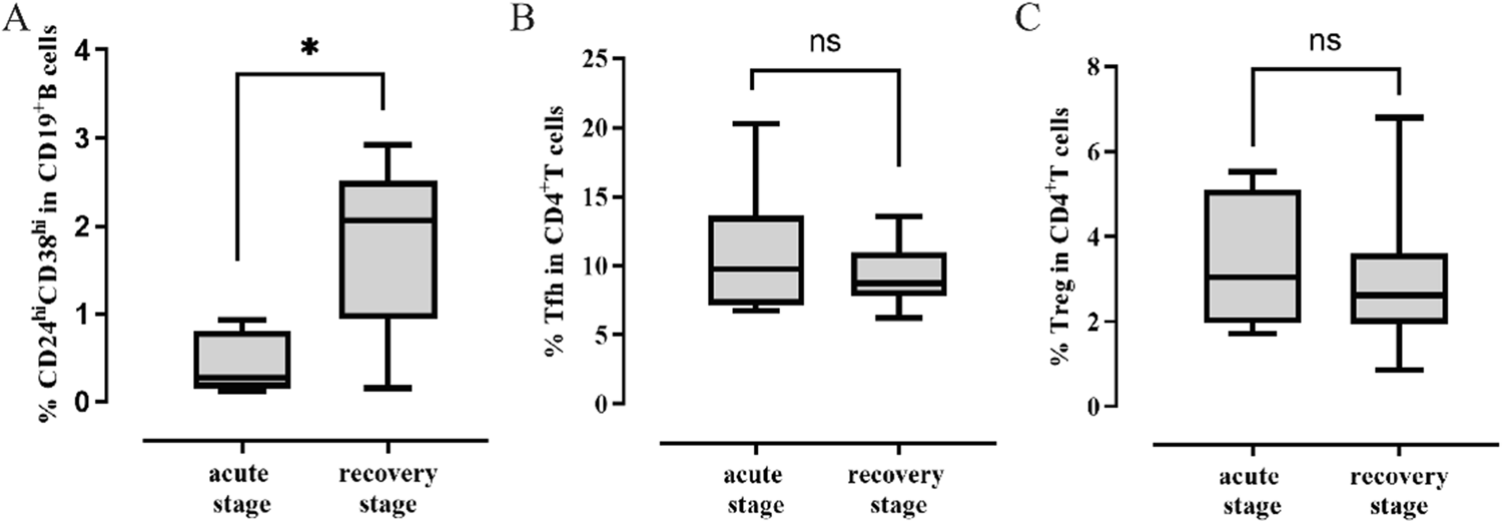
The frequency of CD24^hi^CD38^hi^transtional B cells, Tfh and Tregs in patients with anti-NMDAR encephalitis at different stages. **A** The frequency of CD24^hi^CD38^hi^ transtional B cells at different stages. **B** The frequency of Tfh at different stages. **C** The frequency of Tregs at different stages. Mann–Whitney *U* test was used. ^*^p<0.05

Anti-NMDA encephalitis was categorized as the acute stage and the recovery stage. At the time of sampling, 6 of 16 non-RTX treated anti-NMDAR encephalitis patients were at the acute stage and 10 were at the recovery stage. We compared changes in CD24^hi^CD38^hi^ transitional B cells, Tfh, and Tregs at different stages. As shown in Fig. 3, the frequency of CD24^hi^CD38^hi^ transitional B cells was significantly lower in the acute stage than in the recovery stage (*p*=0.016), but there were no statistically significant differences in Tfh and Tregs frequencies (*p*=0.713 and *p*=0.635, respectively).

### The frequency of CD24^hi^CD38^hi^ transitional B cells is correlated with clinical severity

**Fig. 4:**
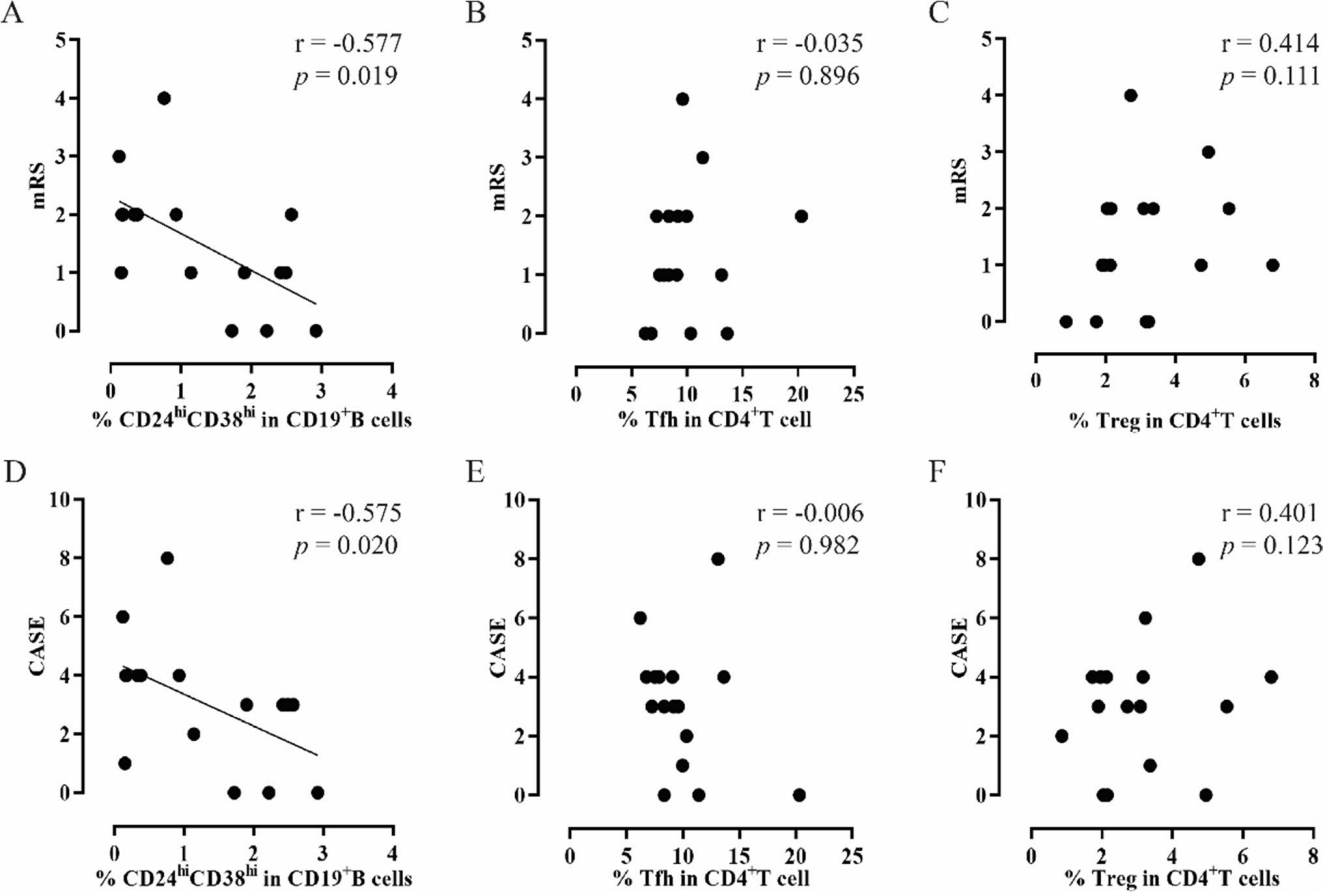
Correlation analysis between CD24^hi^CD38^hi^ transitional B cells, Tfh, Tregs, and neurological status. **A** The correlation analysis between CD24^hi^CD38^hi^ transitional B cells and the mRS. **B** The correlation analysis between Tfh and the mRS. **C** The correlation analysis between Tregs and the mRS. **D** The correlation analysis between CD24^hi^CD38^hi^ transitional B cells and the CASE. **E** The correlation analysis between Tfh and the CASE. **F** The correlation analysis between Tregs and the CASE. Spearman correlation analysis was used

Neurological status was assessed with the mRS and the CASE. We explored the correlation between the above immune cell subsets and neurological status. As shown in Fig. 4, the frequency of CD24^hi^CD38^hi^ transitional B cells was negatively correlated with both the mRS (*r*=−0.577, *p*=0.019) and the CASE (*r*=−0.575, *p*=0.020). However, the frequency of Tfh or Tregs showed no statistically significant correlations neither with the mRS (*r*=−0.035, *p*=0.896 and *r*=0.414, *p*=0.111, respectively) nor with the CASE (*r*=−0.006, *p*=0.982 and *r*=0.401, *p*=0.123, respectively).

### RTX treatment restored CD24^hi^CD38^hi^ transitional B cells

**Fig. 5:**
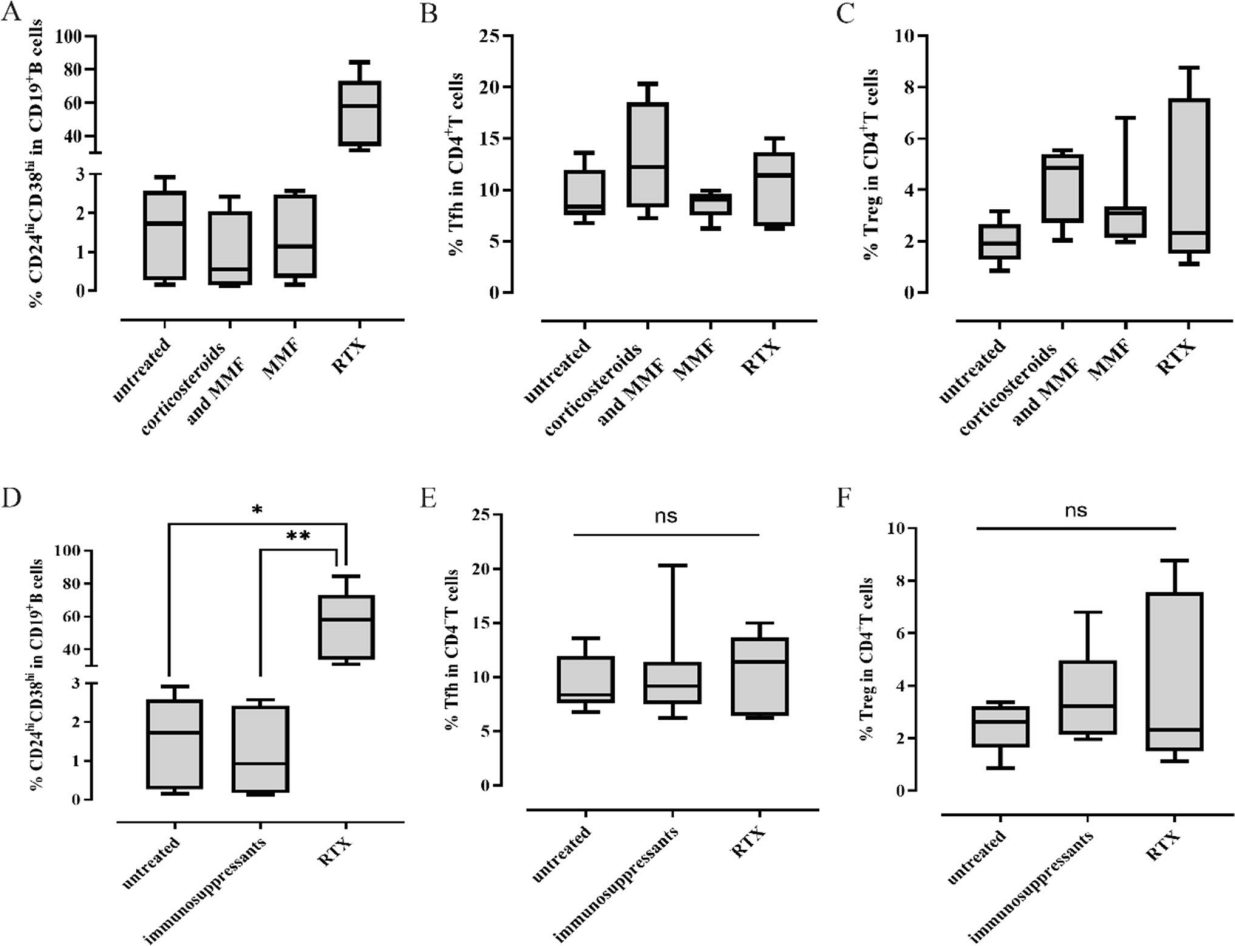
The influence of different treatment regimens on CD24^hi^CD38^hi^ transitional B cells, Tfh and Tregs. **A**, **D** The frequency of CD24^hi^CD38^hi^ transitional B cells in patients treated with different treatment regimens. **B**, **E** The frequency of Tfh in patients treated with different treatment regimens. **C**, **F** The frequency of Treg in patients treated with different treatment regimens. Kruskal-Wallis test with Bonferroni post hoc analyses was used. ^*^p<0.05, ^**^p<0.01

Our results indicated that the frequencies of both CD24^hi^CD38^hi^ transitional B cells and Tregs decreased significantly in patients with anti-NMDAR encephalitis while the frequency of Tfh increased. We next investigated the effect of different treatment regimens on these immune cell subsets. As shown in Fig. 5, the frequency of CD24^hi^CD38^hi^ transitional B cells was similar between patients treated with oral MMF, oral corticosteroids plus MMF, and untreated, but markedly increased following RTX treatment. Whereas, the frequencies of Tregs and Tfh were similar between patients treated with oral MMF, oral corticosteroids plus MMF, RTX, and untreated. Due to a relatively small sample size, we combined patients treated with oral MMF and oral corticosteroids plus MMF into one group (oral immunosuppressants) to analyze. The frequency of CD24^hi^CD38^hi^ transitional B cells at the last follow-up after RTX treatment was significantly higher than those treated with oral immunosuppressants or untreated (*p*=0.004, *p*=0.038, respectively). However, the frequencies of Tregs and Tfh were not significantly different in patients treated with RTX, oral immunosuppressants, or untreated (*p*=0.979, *p*=0.105, respectively).

## Discussion

We explored the frequencies of Bregs, Tfh, and Tregs in patients with anti-NMDAR encephalitis for the first time. Our results demonstrated a significant imbalance in Bregs, Tfh, and Tregs, manifested as a numerical reduction in CD24^hi^CD38^hi^ transitional B cells and Tregs and overexpression of Tfh. The frequency of CD24^hi^CD38^hi^ transitional B cells was significantly lower in the acute stage than in the recovery stage, and was negatively correlated with the mRS and the CASE. Besides, the frequency of CD24^hi^CD38^hi^ transitional B cells at the last follow-up after RTX treatment was significantly higher than those treated with oral immunosuppressants or untreated. As Bregs can suppress the proliferation and differentiation of effector T cells and induce the differentiation of Tregs, we speculate that numerical impairment of CD24^hi^CD38^hi^ transitional B cells leads to over proliferation of Tfh and deficiency of Tregs.

Bregs are essential for the maintenance of tolerance and immune homeostasis, which can suppress the differentiation of effector T cells and skew T cells differentiation in favor of a regulatory phenotype mainly via the expression of IL-10, IL-35, and TGF-β [9]. Numerical or functional impairment of Bregs may lead to overexpression of Tfh and insufficiency of Tregs. Bregs can be induced in response to inflammation at different B cell developmental or activation stages. Almost all B cell subsets can be induced to form Bregs. To date, multiple Bregs subsets with similar functions but different phenotypes have been identified. CD24^hi^CD38^hi^ transitional B cells and CD24^hi^CD27^+^ B10 B cells, which were differentiated from immature B cells and memory B cells respectively, are the two main Bregs subsets in human [23]. Recently, several studies have revealed that the proportion of Bregs was significantly lower in many neurological autoimmune diseases than in healthy individuals, such as MS, NMOSD, MOG-AD, and MG [15–19, 24].

In our study, the frequency of CD24^hi^CD38^hi^ transitional B cells was significantly lower in patients with anti-NMDAR encephalitis compared with healthy controls and noninflammatory neurological disease IIH, while the frequency of CD24^hi^CD27^+^ B10 B cells were comparable. This indicated that CD24^hi^CD38^hi^ transitional B cells were involved in the pathogenesis of anti-NMDAR encephalitis and might be a promising new therapeutic target in the future. The quantitative impairment of Bregs in anti-NMDAR encephalitis was mainly reflected in CD24^hi^CD38^hi^ transitional B cells rather than CD24^hi^CD27^+^ B10 B cells. A potential reason for CD24^hi^CD27^+^ B10 B cells remained unchanged may be related to the fact that there were much larger number of memory B cells in peripheral blood of anti-NMDAR encephalitis patients. In the meantime, it was found that compared with HC and IIH, the frequency of Tregs was significantly lower but the frequency of Tfh was higher. This may be due to the insufficient function of Bregs to suppress the differentiation of effector T cells, and promote Tregs. However, when compared with NMOSD, none of the changes in the above immune cell subsets were significant. Although these two diseases are two completely different diseases, anti-NMDAR encephalitis and NMOSD shared similar circulating lymphocytic subpopulation profiles, which indicated that the changes in Bregs, Tfh, and Tregs were not specific to anti-NMDAR encephalitis, and were also seen in other neurological autoimmune diseases. Furthermore, we found that patients in the acute stage had a lower frequency of CD24^hi^CD38^hi^ transitional B cells than patients in the recovery stage, and the frequency of CD24^hi^CD38^hi^ transitional B cells was negatively correlated with the mRS and the CASE. It indicated that CD24^hi^CD38^hi^ transitional B cells frequency may be a potential indicator to estimate the disease activity and severity.

Immunotherapy is the mainstay of treatment for anti-NMDAR encephalitis. Oral corticosteroids and MMF are commonly used for maintenance immunotherapies, and RTX is a viable option for the treatment of refractory anti-NMDAR encephalitis. We next explored the impacts of different treatment regimens on those immune cell subsets. As we have observed, the frequency of CD24^hi^CD38^hi^ transitional B cells at the last follow-up after RTX treatment was significantly higher than those treated with oral immunosuppressants or untreated, which indicated that CD24^hi^CD38^hi^ transitional B cells were the dominating subset when the B cells began to appear. This is consistent with the results previously reported in NMOSD [24]. Besides, we were surprised to find that patients treated with oral immunosuppressants tended to have a decreased frequency of CD24^hi^CD38^hi^ transitional B cells than untreated patients, although the differences were not statistically significant. This phenomenon can be explained by the facts that corticosteroids can induce B cells apoptosis, and MMF can diminish cell proliferation. It has been proved that all developmental stages of B cells express the glucocorticoid receptor, and dexamethasone can stimulate B cells apoptosis by binding with the glucocorticoid receptor. Immature B cells are more sensitive to the effect of glucocorticoid induced apoptosis [25]. Given that CD24^hi^CD38^hi^ transitional B cells belong to immature B cells, they may be more sensitive to apoptotic effects induced by corticosteroids. MMF prevents cell replication by inhibiting the generation of DNA, and lymphocytes are a relatively specific target of it [26]. As a subset of immature lymphocytes, the proliferation of CD24^hi^CD38^hi^ transitional B cells may be inhibited by MMF. However, oral immunosuppressants and RTX did not affect the frequencies of Tfh and Tregs. This was not consistent with previous findings. Zhao et al. demonstrated that RTX could inhibit increased Tfh in NMOSD, and the frequency of Tfh significantly declined 1 month after RTX treatment [27]. Gwenny et al. found that RTX treatment in primary Sjögren syndrome resulted in a decrease of almost 50% in frequencies of Tfh at 16 weeks after infusion, but the frequency of Tfh returned to baseline levels of patients during B cell repopulation [28]. Our result suggested that the frequencies of Tfh in patients treated with oral immunosuppressants, RTX or untreated were comparable. We presume that this could mainly be due to the different intervals between sampling and the last RTX infusion. The interval between sampling and the last RTX infusion was relatively long in our study, with a median interval of 11 months. Tfh might has already returned to baseline level during B cell repopulation. To date, there have been several studies focused on the impact of RTX treatment on Tregs in systemic autoimmune disorders, but none specifically examined the impact of RTX treatment on Tregs in neurological autoimmune diseases. Constantina et al. found that the percentage of Tregs was not significantly altered following RTX treatment in rheumatoid arthritis [29]. On the contrary, Michelle et al. suggested that the percentage of Tregs weakly increased as early as day 8 after RTX infusion and further increased at 6 months in patients with severe idiopathic membranous nephropathy, further analysis indicated that an increase in Tregs percentage after RTX treatment is observed in responder patients only [30]. Our results showed that the frequency of Tregs did not differ among patients treated with oral immunosuppressants, RTX or untreated. We speculated that the effect of RTX on Tregs may vary, depending on the disease conditions and the response to RTX treatment. Therefore, whether Tregs is affected by RTX treatment in patients with anti-NMDAR encephalitis still needs further exploration.

Our study has several limitations. First, this study was a single-center cross-sectional study with a relatively small sample size, and the longitudinal follow-up was lacking. Second, the follow-up time for measurement of immune cell subsets after RTX treatment was relatively short, and the interval between the last RTX infusion and sampling was not at a fixed time. Third, our study simply suggested that the frequency of CD24^hi^CD38^hi^ transitional B cells was reduced in anti-NMDAR encephalitis, but assessment of their function was lacking. Whether their function is impaired remains to be explored in the future.

In conclusion, we found that the frequencies of CD24^hi^CD38^hi^ transitional B cells and Tregs were decreased while the frequency of Tfh was increased in patients with anti-NMDAR encephalitis. CD24^hi^CD38^hi^ transitional B cells frequency may be a potential indicator to estimate the disease activity and severity.

## Data Availability

The data of the current study are available from the corresponding author on reasonable request.

## References

[1] Guan HZ, Ren HT, Cui LY (2016). Autoimmune encephalitis: an expanding frontier of neuroimmunology. Chin Med J (Engl).

[2] Dalmau J, Armangué T, Planagumà J, Radosevic M, Mannara F, Leypoldt F, Geis C, Lancaster E, Titulaer MJ, Rosenfeld MR, Graus F (2019). An update on anti-NMDA receptor encephalitis for neurologists and psychiatrists: mechanisms and models. The Lancet Neurology.

[3] Dalmau J (2016). NMDA receptor encephalitis and other antibody-mediated disorders of the synapse: the 2016 Cotzias Lecture. Neurology.

[4] Makuch M, Wilson R, Al-Diwani A, Varley J, Kienzler AK, Taylor J, Berretta A, Fowler D, Lennox B, Leite MI, Waters P, Irani SR (2018). N-methyl-D-aspartate receptor antibody production from germinal center reactions: therapeutic implications. Ann Neurol.

[5] Malviya M, Barman S, Golombeck KS, Planaguma J, Mannara F, Strutz-Seebohm N, Wrzos C, Demir F, Baksmeier C, Steckel J, Falk KK, Gross CC, Kovac S, Bonte K, Johnen A, Wandinger KP, Martin-Garcia E, Becker AJ, Elger CE, Klocker N, Wiendl H, Meuth SG, Hartung HP, Seebohm G, Leypoldt F, Maldonado R, Stadelmann C, Dalmau J, Melzer N, Goebels N (2017). NMDAR encephalitis: passive transfer from man to mouse by a recombinant antibody. Ann Clin Transl Neurol.

[6] Kreye J, Wenke NK, Chayka M, Leubner J, Murugan R, Maier N, Jurek B, Ly LT, Brandl D, Rost BR, Stumpf A, Schulz P, Radbruch H, Hauser AE, Pache F, Meisel A, Harms L, Paul F, Dirnagl U, Garner C, Schmitz D, Wardemann H, Prüss H (2016). Human cerebrospinal fluid monoclonal N-methyl-D-aspartate receptor autoantibodies are sufficient for encephalitis pathogenesis. Brain : a journal of neurology.

[7] Liu J, Liu L, Kang W, Peng G, Yu D, Ma Q, Li Y, Zhao Y, Li L, Dai F, Wang J (2020). Cytokines/chemokines: potential biomarkers for non-paraneoplastic anti-N-methyl-D-aspartate receptor encephalitis. Front Neurol.

[8] Liba Z, Kayserova J, Elisak M, Marusic P, Nohejlova H, Hanzalova J, Komarek V, Sediva A (2016). Anti-N-methyl-D-aspartate receptor encephalitis: the clinical course in light of the chemokine and cytokine levels in cerebrospinal fluid. J Neuroinflammation.

[9] Rosser EC, Mauri C (2015). Regulatory B cells: origin, phenotype, and function. Immunity.

[10] Ding T, Su R, Wu R, Xue H, Wang Y, Su R, Gao C, Li X, Wang C (2021). Frontiers of autoantibodies in autoimmune disorders: crosstalk between Tfh/Tfr and regulatory B cells. Front Immunol.

[11] Achour A, Simon Q, Mohr A, Séité JF, Youinou P, Bendaoud B, Ghedira I, Pers JO, Jamin C (2017). Human regulatory B cells control the T (FH) cell response. J Allergy Clin Immunol.

[12] Alhabbab RY, Nova-Lamperti E, Aravena O, Burton HM, Lechler RI, Dorling A, Lombardi G (2019). Regulatory B cells: development, phenotypes, functions, and role in transplantation. Immunol Rev.

[13] Fan X, Lin C, Han J, Jiang X, Zhu J, Jin T (2015). Follicular helper CD4+ T cells in human neuroautoimmune diseases and their animal models. Mediators Inflamm.

[14] Danikowski KM, Jayaraman S, Prabhakar BS (2017). Regulatory T cells in multiple sclerosis and myasthenia gravis. J Neuroinflammation.

[15] Cencioni MT, Ali R, Nicholas R, Muraro PA (2021). Defective CD19+CD24(hi)CD38(hi) transitional B-cell function in patients with relapsing-remitting MS. Mult Scler.

[16] Quan C, Yu H, Qiao J, Xiao B, Zhao G, Wu Z, Li Z, Lu C (2013). Impaired regulatory function and enhanced intrathecal activation of B cells in neuromyelitis optica: distinct from multiple sclerosis. Mult Scler.

[17] Li X, Wang L, Zhou L, ZhangBao J, Miao MZ, Lu C, Lu J, Quan C (2019). The imbalance between regulatory and memory B cells accompanied by an increased number of circulating T-follicular helper cells in MOG-antibody-associated demyelination. Mult Scler Relat Disord.

[18] Lin Y, Chang T, Lin J, Sun C, Wei C, Zhao J, Liu R, Yang K, Li Z (2022). Regulatory B cells are decreased and functionally impaired in myasthenia gravis patients. Front Neurol.

[19] Guerrier T, Labalette M, Launay D, Lee-Chang C, Outteryck O, Lefevre G, Vermersch P, Dubucquoi S, Zephir H (2018). Proinflammatory B-cell profile in the early phases of MS predicts an active disease. Neurol Neuroimmunol Neuroinflamm.

[20] Yang X, Peng J, Huang X, Liu P, Li J, Pan J, Wei Z, Liu J, Chen M, Liu H (2021). Association of circulating follicular helper T cells and serum CXCL13 with neuromyelitis optica spectrum disorders. Front Immunol.

[21] Graus F, Titulaer MJ, Balu R, Benseler S, Bien CG, Cellucci T, Cortese I, Dale RC, Gelfand JM, Geschwind M, Glaser CA, Honnorat J, Hoftberger R, Iizuka T, Irani SR, Lancaster E, Leypoldt F, Pruss H, Rae-Grant A, Reindl M, Rosenfeld MR, Rostasy K, Saiz A, Venkatesan A, Vincent A, Wandinger KP, Waters P, Dalmau J (2016). A clinical approach to diagnosis of autoimmune encephalitis. Lancet Neurol.

[22] Kayser MS, Dalmau J (2011). Anti-NMDA receptor encephalitis in psychiatry. Curr Psychiatry Rev.

[23] Oleinika K, Mauri C, Salama AD (2019). Effector and regulatory B cells in immune-mediated kidney disease. Nat Rev Nephrol.

[24] Quan C, ZhangBao J, Lu J, Zhao C, Cai T, Wang B, Yu H, Qiao J, Lu C (2015). The immune balance between memory and regulatory B cells in NMO and the changes of the balance after methylprednisolone or rituximab therapy. Journal of neuroimmunology.

[25] Gruver-Yates AL, Quinn MA, Cidlowski JA (2014). Analysis of glucocorticoid receptors and their apoptotic response to dexamethasone in male murine B cells during development. Endocrinology.

[26] Broen JCA, van Laar JM (2020). Mycophenolate mofetil, azathioprine and tacrolimus: mechanisms in rheumatology. Nat Rev Rheumatol.

[27] Zhao C, Li HZ, Zhao DD, Ma C, Wu F, Bai YN, Zhang M, Li ZY, Guo J (2017). Increased circulating T follicular helper cells are inhibited by rituximab in neuromyelitis optica spectrum disorder. Front Neurol.

[28] Verstappen GM, Kroese FG, Meiners PM, Corneth OB, Huitema MG, Haacke EA, van der Vegt B, Arends S, Vissink A, Bootsma H, Abdulahad WH (2017). B cell depletion therapy normalizes circulating follicular Th cells in primary Sjogren syndrome. J Rheumatol.

[29] Bounia CA, Liossis SC (2021). B cell depletion treatment decreases Th17 cells in patients with rheumatoid arthritis. Clin Immunol.

[30] Rosenzwajg M, Languille E, Debiec H, Hygino J, Dahan K, Simon T, Klatzmann D, Ronco P (2017). B- and T-cell subpopulations in patients with severe idiopathic membranous nephropathy may predict an early response to rituximab. Kidney Int.

